# A New Mathematical Model for the Interpretation of Translational Research Evaluating Six CTLA-4 Polymorphisms in High-Risk Melanoma Patients Receiving Adjuvant Interferon

**DOI:** 10.1371/journal.pone.0086375

**Published:** 2014-01-27

**Authors:** Petr Pancoska, John M. Kirkwood, Spyros Bouros, Maria Spyropoulou-Vlachou, Eirini Pectasides, Dimosthenis Tsoutsos, Aristidis Polyzos, Christos Markopoulos, Petros Panagiotou, Ourania Castana, Dimitrios Bafaloukos, George Fountzilas, Helen Gogas

**Affiliations:** 1 Department of Medicine, Center for Clinical Pharmacology, Center for Craniofacial and Dental Genetics, University of Pittsburgh, Pittsburgh, Pennsylvania, United States of America; 2 Department of Medicine, Hillman Cancer Center, University of Pittsburgh Cancer Institute, Pittsburgh, Pennsylvania, United States of America; 3 1^st^ Department of Medicine, Medical School, University of Athens, Athens, Greece; 4 Department of Immunology, National Tissue Typing Center, General Hospital of Athens, Athens, Greece; 5 Department of Plastic Surgery and Microsurgery, G. Gennimatas General Hospital of Athens, Athens, Greece; 6 Department of Plastic Surgery, Evangelismos General Hospital of Athens, Athens, Greece; 7 Hellenic Cooperative Oncology Group, Data Office, Athens, Greece; Centers for Disease Control and Prevention, United States of America

## Abstract

Adjuvant therapy of stage IIB/III melanoma with interferon reduces relapse and mortality by up to 33% but is accompanied by toxicity-related complications. Polymorphisms of the CTLA-4 gene associated with autoimmune diseases could help in identifying interferon treatment benefits. We previously genotyped 286 melanoma patients and 288 healthy (unrelated) individuals for six CTLA-4 polymorphisms (SNP). Previous analyses found no significant differences between the distributions of CTLA-4 polymorphisms in the melanoma population vs. controls, no significant difference in relapse free and overall survivals among patients and no correlation between autoimmunity and specific alleles. We report new analysis of these CTLA-4 genetic profiles, using Network Phenotyping Strategy (NPS). It is graph-theory based method, analyzing the SNP patterns. Application of NPS on CTLA-4 polymorphism captures allele relationship pattern for every patient into 6-partite mathematical graph P. Graphs P are combined into weighted 6-partite graph S, which subsequently decomposed into reference relationship profiles (RRP). Finally, every individual CTLA-4 genotype pattern is characterized by the graph distances of P from eight identified RRP's. RRP's are subgraphs of S, collecting equally frequent binary allele co-occurrences in all studied loci. If S topology represents the genetic “dominant model”, the RRP's and their characteristic frequencies are identical to expectation-maximization derived haplotypes and maximal likelihood estimates of their frequencies. The graph-representation allows showing that patient CTLA-4 haplotypes are uniquely different from the controls by absence of specific SNP combinations. New function-related insight is derived when the 6-partite graph reflects allelic state of CTLA-4. We found that we can use differences between individual P and specific RRPs to identify patient subpopulations with clearly different polymorphic patterns relatively to controls as well as to identify patients with significantly different survival.

## Introduction

Adjuvant therapy of patients with stage IIB/III melanoma (high-risk) with interferon was approved by FDA (United States Food and Drug administration) and subsequently by regulatory authorities worldwide [1]. Despite the ability of this regimen to reduce relapse and mortality by up to 33% [2]–[4] acceptance has been limited due to toxicity of this regimen. Attempts to identify the subset of patients destined to benefit from adjuvant treatment with IFNα-2b have failed to discover clinical or demographic features of the patient population that are capable of predicting the benefit from high dose interferon (HDI) therapy. Correlative studies have been undertaken over the years, demonstrating a variety of immunological responses subsequent to therapy [5], [6].

We recently published a paper in which six CTLA-4 polymorphisms were evaluated in a cohort of patients treated with adjuvant interferon [7]. The human CTLA-4 gene is located on chromosome 2q33, in a region that is associated with susceptibility for autoimmune disease [8] and multiple polymorphisms of the CTLA-4 gene have been found to be associated with susceptibility to autoimmune diseases (e.g. the GG allele of the +49 AG polymorphism is associated with decreased expression of CTLA-4 upon T-cell activation and thus a higher proliferation of T-cells) [9]–[12].

We genotyped DNA isolated from the peripheral blood of a total of 286 patients with high-risk melanoma who participated in a prospective multicenter randomized phase III trial of adjuvant interferon and a panel of 288 randomly selected healthy unrelated Greek individuals from the Donor Marrow Registry of the National Tissue Typing Center, Athens, Greece that served as a control population for 6 CTLA4-SNPs of potential interest – namely CT 60, AG 49, CT 318, JO 27, JO 30 and JO 31. CT 318 is located within the promoter region of the CTLA-4 gene, A/G49 is located at exon 1, while the rest of the SNPs tested are located at the 3′ untranslated region of CTLA-4.

High levels of association among the different polymorphisms were found (Fisher's exact *p* value<0.001 for all associations). Genotypes corresponding to the six CTLA-4 polymorphisms did not significantly deviate from the Hardy-Weinberg equilibrium. This indicates significant linkage disequilibrium among the six polymorphisms. We analyzed the segregation pattern of CT 318, AG 49, CT 60, JO 27, JO 30, JO 31 SNPs on 572 chromosomes and identified 5 major haplotypes. No statistically significant differences for relapse free survival or overall survival were found for the presence of each of the 3 most common haplotypes. When the respective polymorphisms were considered separately for outcome analysis by the allele status, or when the three most significant haplotypes were considered, two results emerged:

1. No significant differences were found between the distributions of CTLA-4 polymorphisms in the melanoma population compared with healthy controls.
2. Relapse free survival (RFS) and overall survival (OS) did not differ significantly among patients with the alleles represented by these polymorphisms. No correlation between autoimmunity and specific alleles was evident.

The results reported in the original paper [7] considered “dominant model” in which both homozygous and heterozygous copies of the six assayed SNP loci were assumed to have similar effect on altering the CTLA-4 function.

We use the original experimental genotyping results on CTLA-4 genotype profile as risk factor as the basis for the new analysis designed and undertaken in this paper. A novel general method of pattern analysis, referred below as network phenotyping strategy (NPS), was introduced for integrative, relationship-based analysis of clinical data [13]–[15]. In the particular application described in this paper, NPS replaces analysis of CTLA-4 individual alleles and allele frequencies by the analysis of relationships between CTLA-4 alleles for every individual in the study. NPS solves two types of problems: First, the “power” problem is addressed, which complicates the use of methods that approach such complete-relationship based analysis by using large number of interaction terms, which requires large number of subjects for informative statistical analyses. NPS captures instead the actual polymorphism relationship patterns cumulatively into special mathematical graphs. Second, NPS-processing of genotyping data eliminates using a priori hypothesis about the role of homozygous and heterozygous allelic forms of the studied genomic variants. Graph-theory based representation of the genotyping results through NPS provides unifying quantitative representation of the complete status of all CTLA-4 variants individually for each patient. In our CTLA-4 genotyping data, we thus do not analyze independent interrelationships among the 153 possible combinations of AA, AB and BB alleles of the six studied CTLA-4 polymorphism. Instead, we take advantage of the fact that all those 153 relationships can be captured in a single relationship pattern graph. A path in this graph then encodes the actual complete experimental CTLA-4 genotyping results for every studied subject. In this way, the complete information about all allele relationships for an individual is captured by a single mathematical object. An important property of the NPS analysis is that, from the collection of all individual SNP relationship patterns, we can additionally compute (in a deterministic, non-statistical way) a framework of directly clinically and functionally interpretable reference relationship profiles (RRP). These RRP's represent “landmarks” in the (multidimensional) clinical/genotypic relationship data space. The clinical significance of the RRP landmarks is then measurable in terms of how many patients have close (but not necessarily identical) personal CTLA-4 genotype relationship patterns to those “landmarks”. For the concrete example of CTLA-4 polymorphisms studied in this paper, RRP's represent limiting characterization of the CTLA-4 SNP co-occurrence patterns. The main advantage of the NPS approach is its identification of any significant heterogeneity that might be captured in the data from the clinical, or in this case the CTLA-4 based immune regulation mechanism that we focused upon in this study of subjects with and without melanoma. These results can be then used in designing follow-up clinical studies.

## Materials and Methods

### Materials

Genotyping of DNA isolated from the peripheral blood of a total of 286 patients with melanoma and a panel of 288 randomly selected healthy unrelated Greek individuals that served as a control population was described in detail previously. Details of the institutional review board and ethics committee approval have previously been published [16]. Six CTLA-4 SNPs were studied, namely CT 60 (rs3087243), AG 49 (rs231775), CT 318 (rs5742909), JO 27 (rs11571297), JO 30 (rs7565213) and JO 31 (rs11571302). CT 318 is located within the promoter region of the CTLA-4 gene, A/G49 is located at exon 1, while the rest of the SNPs tested are located at the 3′ untranslated region of CTLA-4.

### Methods

#### Characterization of personal CTLA-4 genotype relationship pattern by 6-partite graphs: Identifying the part of the study data in which we have maximal information to extract additional components of information

**Figure 1:**
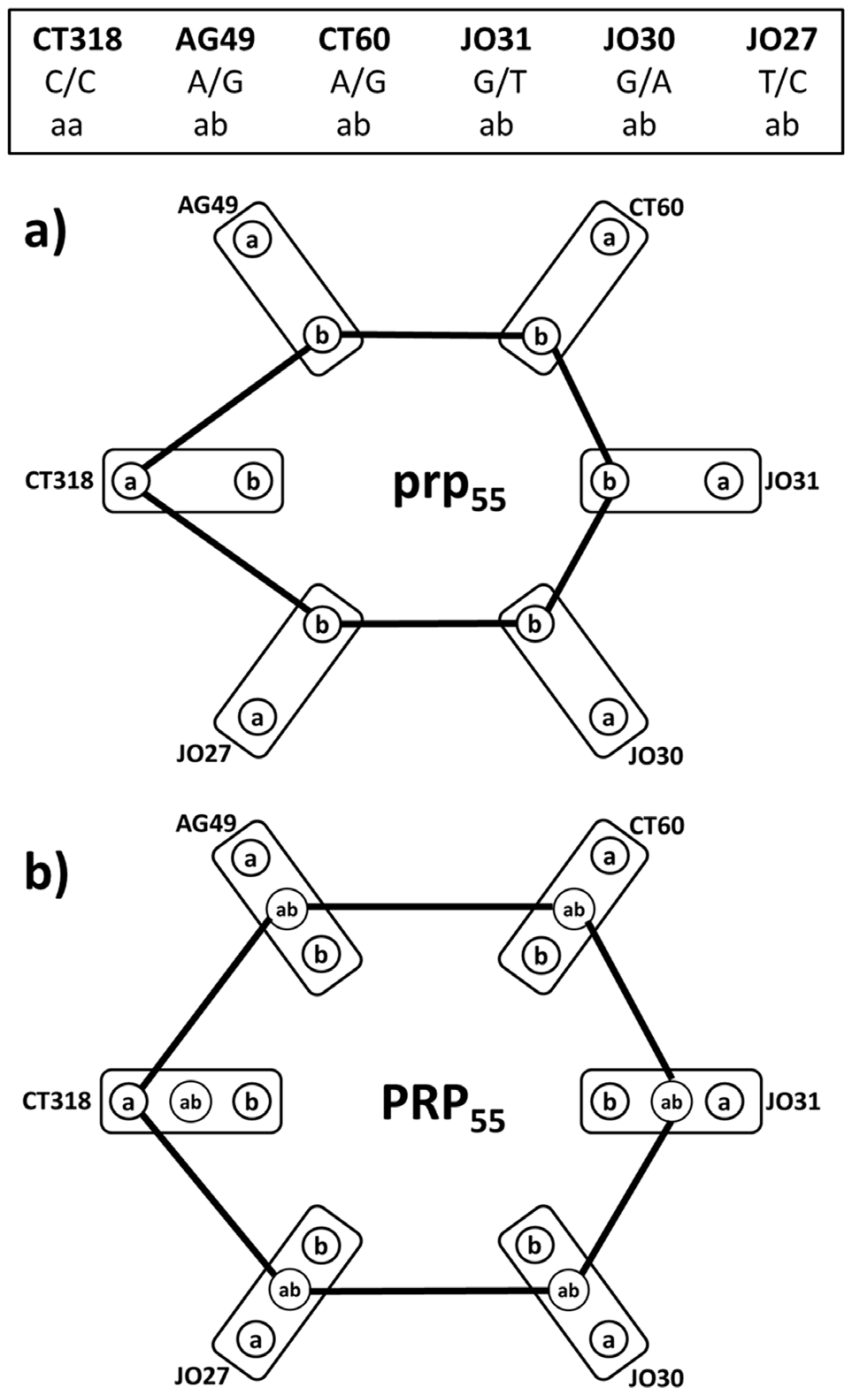
Example how experimentally determined CTLA-4 genotype (top panel) for a patient (id = 55) is transformed into a) *prp* graph and b) *PRP* graph. **a**-major allele, **b**-minor allele, **ab**-heterozygous allele status vertices. Each SNP is represented by a graph partition (rectangles), identified by the SNP code. Lines – graph edges, representing the co-occurrences of all alleles in the patient's CTLA-4 genotype.

We present two levels of CTLA-4 genotype analysis. In the first one, we do not distinguish between homozygous or heterozygous status of the six alleles. In the second one, we will expand the genotype characterization using the known zygosity of the six SNP's. **Fig. 1a** shows how an observed CTLA-4 genotype for one patient may be represented by a 6-partite graph that will be called a personal relationship profile ***prp***, which we use for the purpose of the first analysis type, considering the major/minor allele relationships only (**Fig. 1a**). In **Fig. 1b** we define the type of personal relationship profile, for which symbol ***PRP*** is used to emphasize that allele relationships include observed allele zygosity. In both these representations, each assayed SNP is represented by one of six partitions in the ***prp*** or ***PRP***. Each partition contains two or three vertices, representing the allele for a given polymorphism (**a** =  major allele, **b** =  minor allele in ***prp***, **a** =  major homozygous, **ab** =  heterozygous, **b** =  minor homozygous allele in ***PRP***). Edges in both graphs connect only those vertices in different partitions that represent observed (genotyped) alleles in the two different polymorphic sites. The complete CTLA-4 genotype profile for an individual is then a collection of edge-connected vertices in ***prp*** **/** ***PRP***, forming a cycle in ***prp*** **/** ***PRP***. Because the edges in ***prp*** **/** ***PRP*** represent relationships between the allelic states of the studied SNP's, there is clear meaning for each segment of the CTLA-4 genotype illustrated in the hexagonal cycle. We can understand these lines in as conditional relationships of type “if AG49 contains minor allele then CT60 contains also minor allele and J031 contains minor allele and then …. “. Note that the experimentally defined cycle in e.g. ***prp*** represents not only the pair wise conditional relationships shown by lines such as (AG49  = **b** when CT60  = **b**), but also all other co-occurrences such as (AG49  = **b** when JO30  = **b**) etc. The ***prp*** **/** ***PRP*** cycle representation of the CTLA-4 SNP allele status co-occurrences is the simplest one capturing all co-occurrence relationships while maintaining convenient mathematical simplicity.

#### Collective characterization of CTLA-4 genotype profile distribution in a cohort by cumulative weighted 6-partite graph *G*

While ***PRP***'s are exact “qualitative” representation of the studied polymorphism relationship patterns in CTLA-4, we need to convert this qualitative information into quantitative characterization of these individual relationship patterns. It has been shown by exact mathematical theorem [17] that the maximal quantitative information captured by graphs is obtained when ***PRP***'s are compared to one another in graphs of the same type, which we call reference relationship patterns (***RRP***). Therefore, the next step of NPS transformation of the CTLA-4 polymorphism relationship patterns into quantitative descriptors is to use the actual data to derive the 6-partite graphs, representing the ***RRP***'s we need.

For this purpose, the individual ***prp*** or ***PRP*** graphs, describing the SNP co-occurrences for all subjects were assembled into cumulative 6-partite “study graphs” ***g*** and ***G***. By adding every individual patient CTLA-4 genotype profile representation ***prp*** to the cumulative ***g*** graph, the weightings of every edge in ***g*** is increased by one, and similarly but independently for PRP's and ***G*** **.** As a consequence of this construction, these ***g*** and ***G*** graphs will have weighted edges defined by the co-occurrence frequencies of all SNP pairs. The distribution of all individual CTLA-4 genotype profiles in case cohort is now represented by graph ***g***.

**Figure 2:**
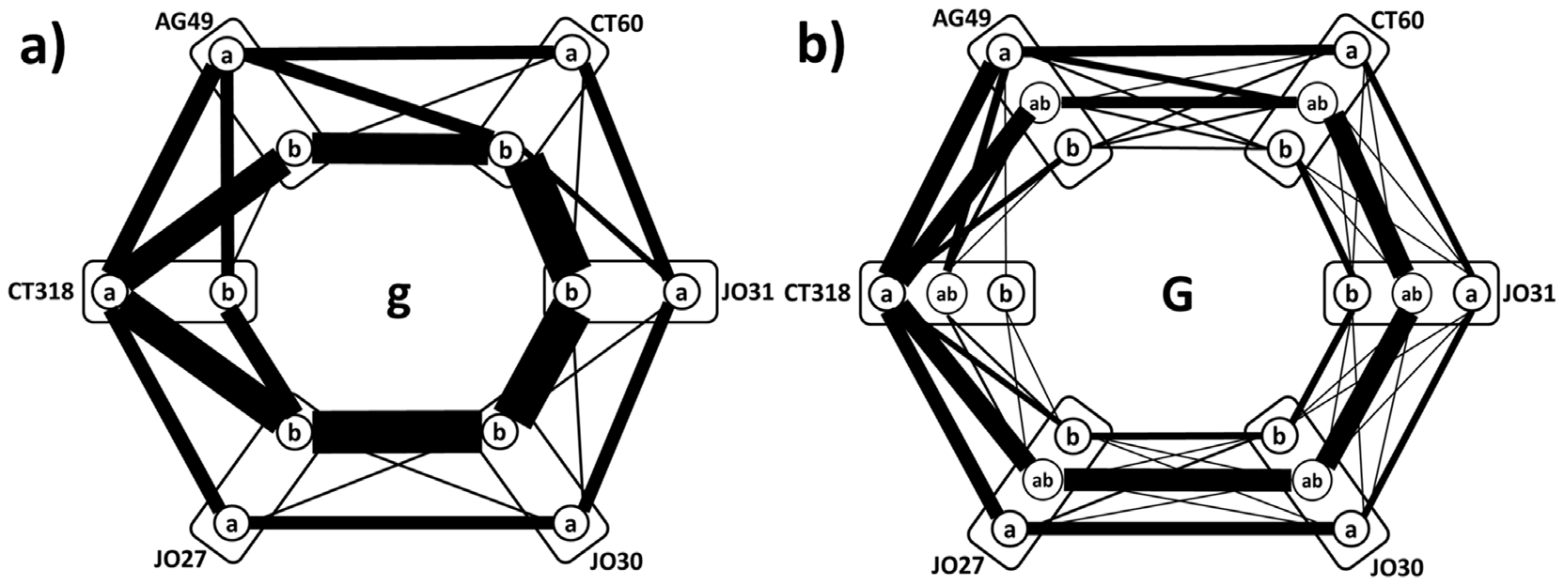
Study graphs *g*(a) and *G* (b) constructed as union of all *prp*'s (*g*) or *PRP*'s (*G*). Symbols as in Fig. 1, thickness of edges in ***g*** and ***G*** are proportional to co-occurrence frequencies of respective SNP pairs, connected by the edge.

In **Fig. 2**, the relative edge weights, resulting from adding all individual case graphs ***prp*** and ***PRP*** to ***g*** and ***G***, respectively, are graphically represented by the variable relative thickness of the edge lines. By converting these edge counts to frequencies, statistical interpretation of the basic vertex-weighted edge-vertex (**a**–**b**), (**a**–**a**), (**b**–**a**) and (**b**–**b**) motifs in study graphs is obtained**.** The weights of study graph edges connecting, for example, the major and minor allele vertices in the AG49 and CT60 partitions define the estimates of the following conditional probabilities:
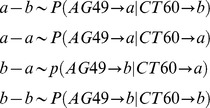



#### In the next step, the complete sets of reference relationship patterns for CTLA-4 genotypes in both study graphs g and G are identified and in case of g identified as haplotypes

Haplotype is defined as a series of polymorphisms in CTLA-4 genotype profile that are co-occurring with identical probabilities, *P*(1)∼*P*(2)∼ … ∼*P*(6). Using the conditional probability interpretation of edges in the study graphs shown in above example, we can derive from the Bayes' theorem, that if sub-graphs of the study graph with equal weights (co-occurrence frequency components) are found, the condition of *P*(1)∼*P*(2)∼ … ∼ *P*(6) is automatically fulfilled. Thus, in our representation, a complete set of haplotypes is represented by all ***RRP*** cycle subgraphs with equal weights of all edges, which can be found in ***g*** or ***G*** by “greedy” algorithm (File S1 and Figure S1).

For validation of this study graph-based approach to haplotype identification, established procedures were additionally used where the maximum likelihood estimates of haplotype frequencies given a multi-locus sample of genetic marker genotypes [3 different genotypes of the 6 polymorphisms] were generated using the expectation-maximization (EM) algorithm under the assumption of Hardy-Weinberg equilibrium (HWE). Linkage disequilibrium was explored for each pair of the 6 polymorphisms (PROC HAPLOTYPE). SAS 9.1 (SAS Institute Inc., Cary, NC, USA), was used for the statistical analysis (reported in [7]).

#### Quantitative characterization of differences of personal CTLA-4 genotype profiles *prp* and *PRP* from haplotypes, represented by *rrp*'s and *RRP*'s

**Figure 3:**
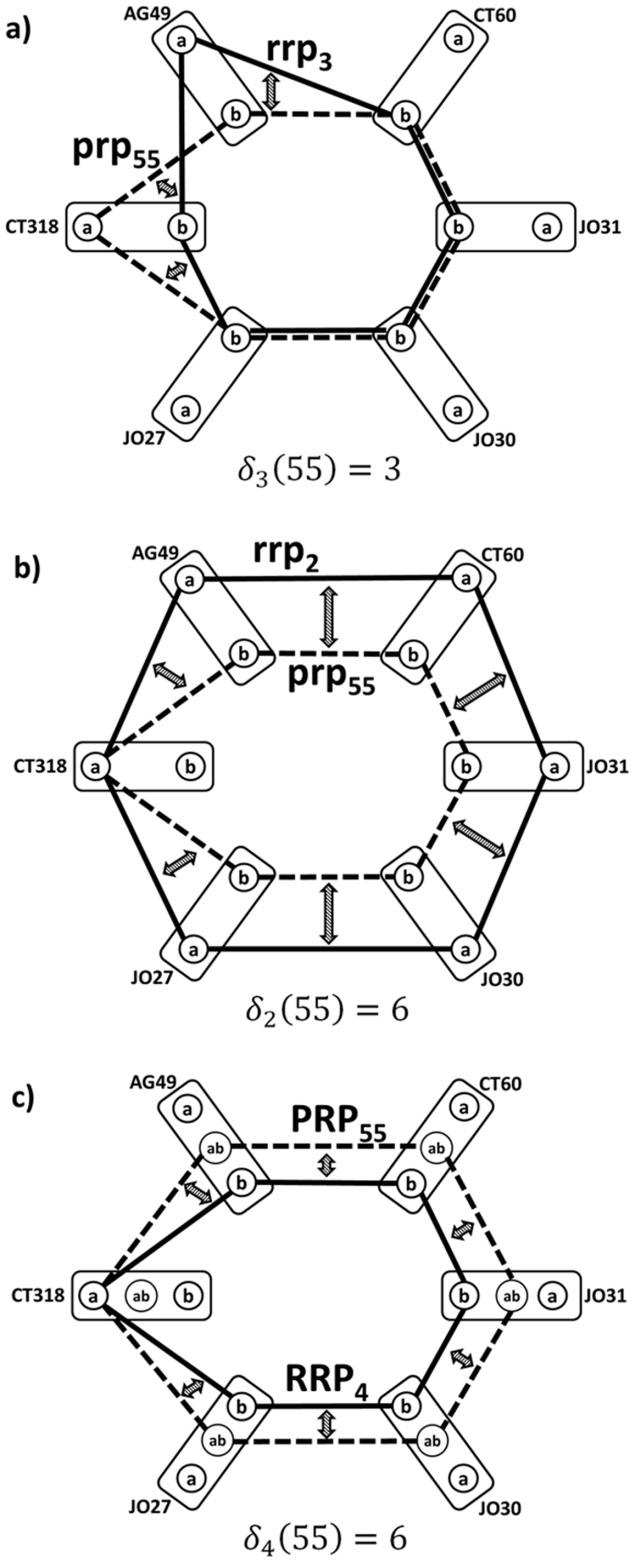
Three examples showing how elements of distance vectors 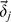
are computed for the same patient #55. In all figures, ***prp*** **(** ***RRP*** in **c)**) for this patient  =  dashed lines, ***rrp***'s (or ***RRP*** in **c)**)  =  solid lines. Double arrows indicate mismatch in SNP co-occurrences. Elements of 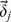 are sums of these mismatches (in computations, we add negative sign to make identity (zero mismatches) mathematically largest). **a,b)** Comparison of patient's genotype to the second and third reference SNP relationship patterns ***rrp_3_*** and ***rrp_2_***_**.**_ **c)** Comparison of patient's genotype to the 4^th^ reference SNP relationship pattern ***RRP_4_*** **.**

For the quantification of the graph-graph distances between individual patient relationship patterns and haplotype-reference relationship patterns, we use the mathematical results of [17], [18], showing that one of the possible definitions of graph-graph distances with all necessary mathematical properties is obtained simply by counting the number of edge mismatches between the two graphs, as is shown by example in Fig. 3. As the result, with haplotype decomposition of study graph **g** resulting in 8 haplotype components, each subject (**j**) is characterized by an 8-element vector 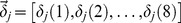 of eight distances of the personal CTLA-4 genotype profile from compositions of all 8 respective haplotypes identified. Difference vectors 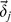 were computed for all patients and controls using a) the control cohort-defined haplotypes and b) the case cohort-defined haplotypes.

#### Developing the hierarchical model for differentiating between healthy controls and melanoma cases using the CTLA-4 based personal genotype profiles from haplotypes

Weka package (v. 3-6-6) implementation of J48 pruned tree algorithm was used to construct optimal model recognizing the controls from cases using personal difference vectors 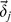. Ten-fold cross-validation was used and characterized the model quality by confusion matrices and ROC parameters.

## Results

**Figure 4:**
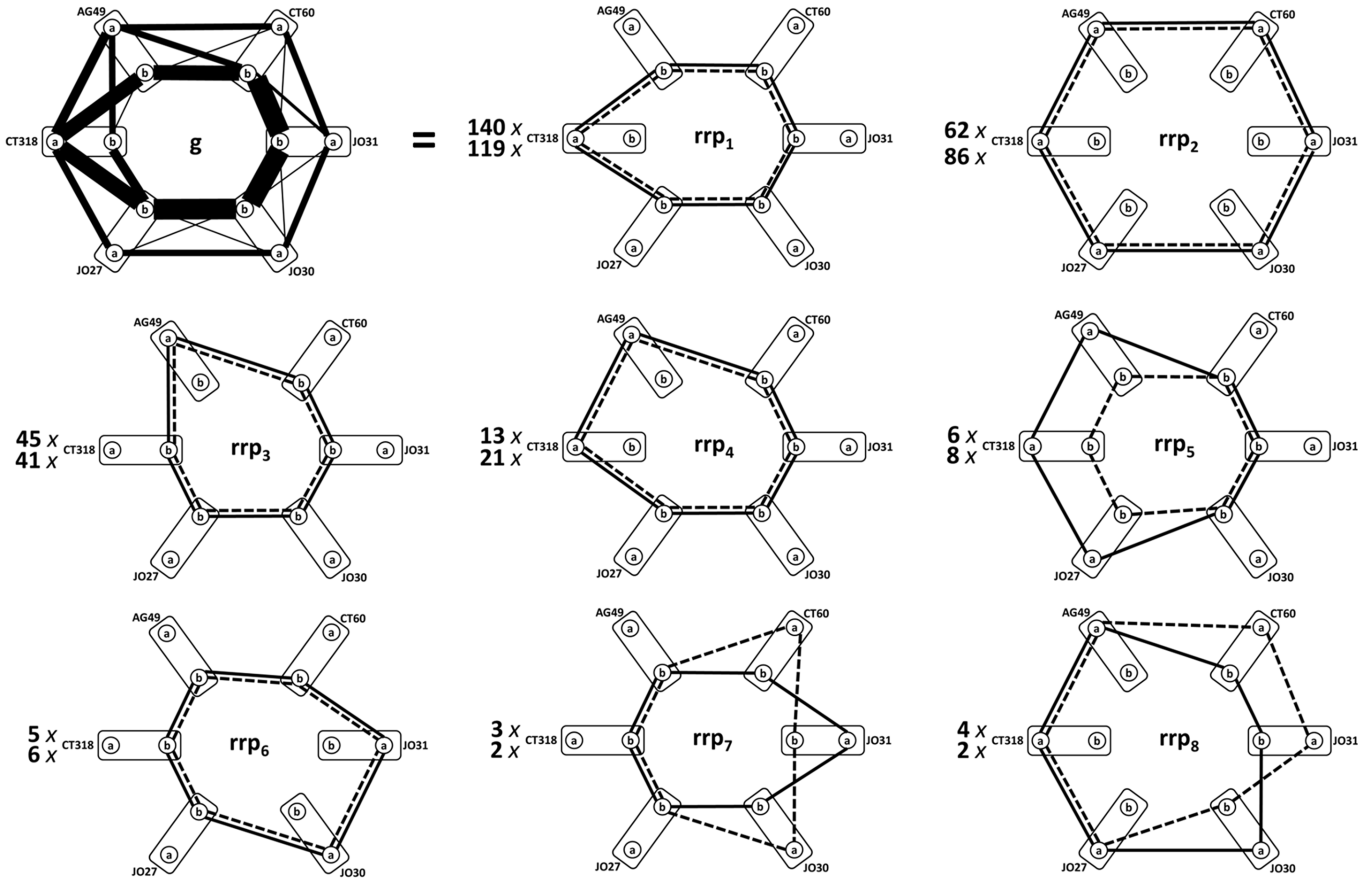
Decomposition of study graphs *g* (picture represents both cases and control subcohorts) into *rrp*'s 1–8. Case ***rrp***'s are shown by solid, control by dashed edges. Coefficients show the multiplicities of respective ***rrp***'s in the ***g***-decompositions (top  =  case graph, bottom  =  control graph). Symbols as in Fig. 1.

Fig. 4 shows decomposition of the ***g*** graphs for healthy controls (**Fig. 2a**) and melanoma cases (**Fig.** **2b**) into component cycles ***rrp_i_*** *,* representing the haplotypes derived from individual genotyped profiles, containing CT60 (rs3087243), AG49 (rs231775), CT318 (rs5742909), JO27 (rs11571297), JO30 (rs7565213) and JO31 (rs11571302) SNPs.

Decomposing the 6-partite graph ***G*** constructed with explicit 3 allele states resulted in 20 ***RRP***. We then computed a 20-component vector of distances 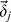 for every personal CTLA-4 genotype relationship pattern from all 20 ***RRP***'s.

### Results for study graph *g*

In both cohorts, the respective ***g*** graphs were decomposed into 8 cycles ***rrp_i_*** (***i*** = 1…8). Interestingly (and importantly) the three haplotype graphs with the largest frequency were identical for control and case cohorts. **Table** **1** shows that our ***g***-based graph algorithm also identified the same dominant haplotypes and comparable frequencies of occurrence as the statistical algorithm in (PROC HAPLOTYPE). SAS 9.1 (SAS Institute Inc., Cary, NC, USA).

**Table 1:** CTLA-4 most frequent haplotypes identified by two methods – using HAPLOTYPE procedure in SAS (ref. [5]) and from multiplicity of ***rrp***'s in decomposition of study graph ***g***.

AG49	CT60	CT318	JO27	JO30	JO31	CTLA-4 haplotype frequencies from ref.[5]		
rs231775	rs3087243	rs5742909	rs11571297	rs7565213	rs11571302	Frequency [%]	Standard Error	Haplotype frequency using *rrp*'s – this work [%]
A	A	C	C	A	T	46.99	2.089	45.7
G	G	C	T	G	G	29.34	1.91	23.0
A	G	T	T	G	G	9.77	1.24	10.2
A	G	C	T	G	G	6.49	1.031	6.0
A	G	C	C	A	T	2.81	0.69	2.5

A unique feature of this approach in comparison to the analysis of differences in haplotype frequencies that were tested in our previous paper is that we can quantitatively characterize the difference of the individual genotype profile from “averaged” CTLA-4 haplotype profiles. **Fig.** **3** demonstrates the meaning of the differences. In this example, patient's **P55** CTLA-4 genotype profile captured into ***ppr***(**55**) matches the composition of the graph representation of haplotype ***rrp_3_*** in just three edges, thus the 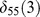 is 3. In the second example, CTLA-4 genotype profile of the same patient is compared to ***C_2_*** haplotype. Here no edges in ***ppr***(**55**) coincide with those of ***rrp_2_***, thus the 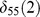
is 6. This is the example of maximal difference between any haplotype subgraph ***rrp_i_*** and individual CTLA-4 genotype profile **prp**(***j***) that can be found in ***g***.

**Figure 5:**
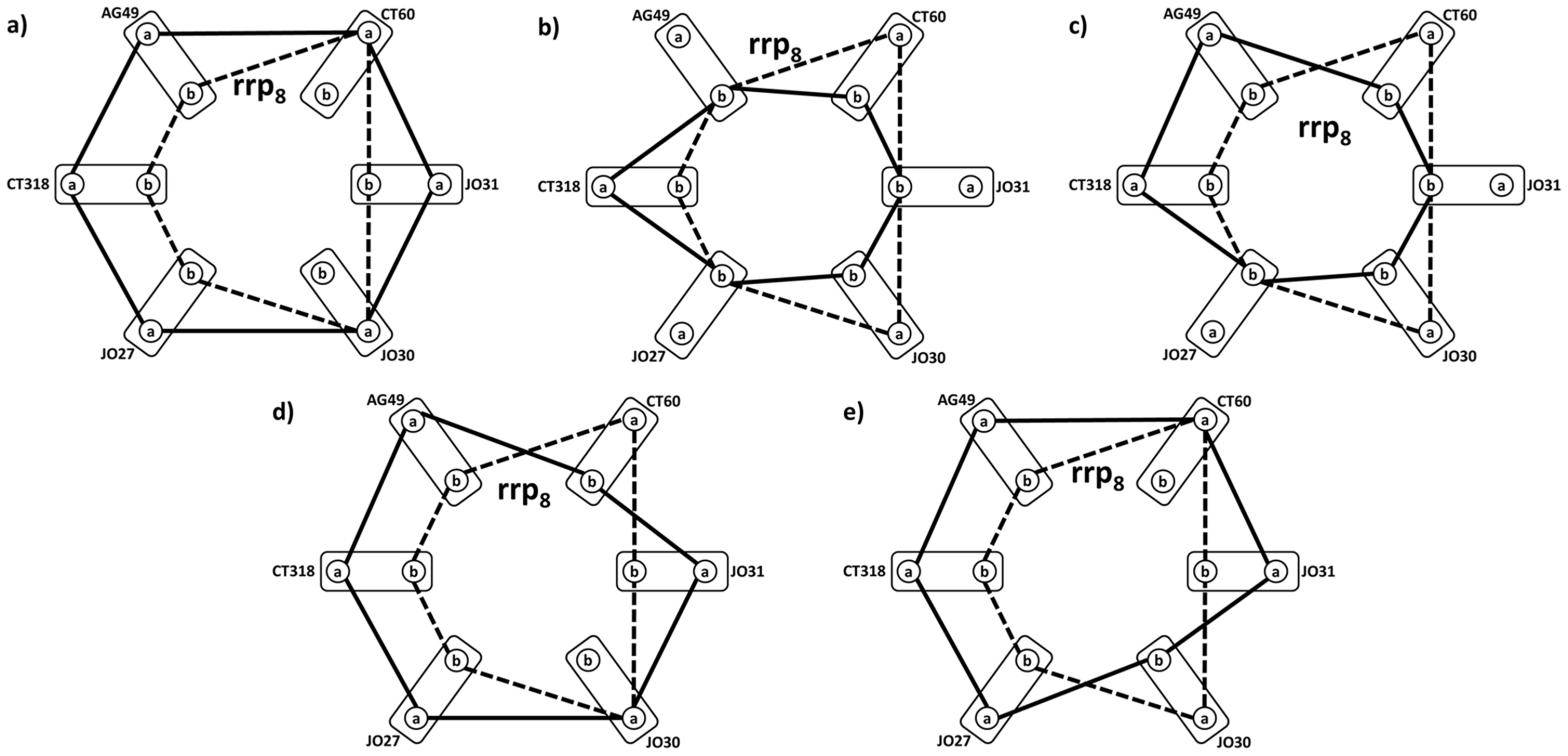
Case-control discrimination by “missing” CTLA-4 genotype reference profile *rrp_8_* (dashed lines in all figures). Solid lines in schemes **a**) – **e**) show five ***prp*** CTLA-4 genotype profiles, found exclusively for 219 (77%) patients identified from the complete case cohort by condition that their ***prp*** have maximal possible distance from the ***rrp_8_***. Symbols as in Fig. 1.

**Fig.** **5** explains the main finding of this paper. Top level of CTLA-4 genotype profile-based differentiation between cases and controls is related to SNP pattern ***rrp_8_***  =  (**bbabab**) for (CT318-AG49-CT60-JO30-JO27) cycle (see **Fig.** **4** and **5**). 77% of melanoma cases (219 patients) are recognized from healthy controls by the ABSENCE of the ***rrp_8_***  =  (**bbabab**) allele pattern for (CT318-AG49-CT60-JO30-JO27) SNP cycle. By surveying all 219 CTLA-4 individual genotype profiles for patients with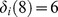 it was found that all have one of the five co-occurring patterns, shown by solid line cycles in **Fig.** **5a–e**. By overlaying the ***rrp_8_***  =  (**bbabab**) case-control differentiating pattern (dashed line cycles) over these actual case-specific genotype profiles it is shown that the ***rrp_8_*** pattern does not share any relationship with these 5 melanoma-characteristic CTLA-4 SNP co-occurrence patterns, indicating the possibility of disease risk identification not by presence, but actually absence of specific genotype profile. Graph mathematics opens the previously overlooked half of the marker identification “Universe” – allowing us to study invariants (such as our personalized differences of CTLA-4 genotype profiles from the haplotype reference) and identifying multiple SNP relationship patterns that share certain properties (simultaneous presence or absence of a specific combination of parameters).

### Results for study graph *G*

The first information that comes from NPS-graph of the CTLA-4 genotype considering the “collective allelic status” of all six studied SNP's (see **Fig.** **2b**). With exception of CT318, there is a strong preference for “allelic state conservation” in all remaining loci: most frequent allelic status for (CT318-AG49-CT60-JO30-JO27) SNP is heterozygous (**a-ab-ab-ab-ab-ab**) profile, the second most frequent is profile with all homozygous wild-type alleles, followed by homozygous (**a-b-b-b-b-b**) profile. Subjects who had mixed type zygosity (**a-ab-a-b-a**… etc) CTLA-4 profiles are minority in this study cohort. Because it was known that there are no characteristic simple CTLA-4 genotype patterns that would differentiate healthy controls from melanoma cases, we instead looked for differences in distances from the all possible ***RRP_1_−RRP_20_*** pairs that would maximize the separation of the two sub-cohorts. The motivation for this approach is as follows: The pattern-based genotype data transformation captures more details of inter-subject differences in genetic status of CTLA-4 than can be captured by any conventional analytical approach. This information enhancement can be further increased by explicitly considering the actual allele statuses. as discussed above, the identification of the clinically relevant context relationship CTLA-4 genotype pattern is obtained by looking for a higher frequency of patients or controls with smaller distances from selected ***RRP***'***s***, relative to others.

**Figure 6:**
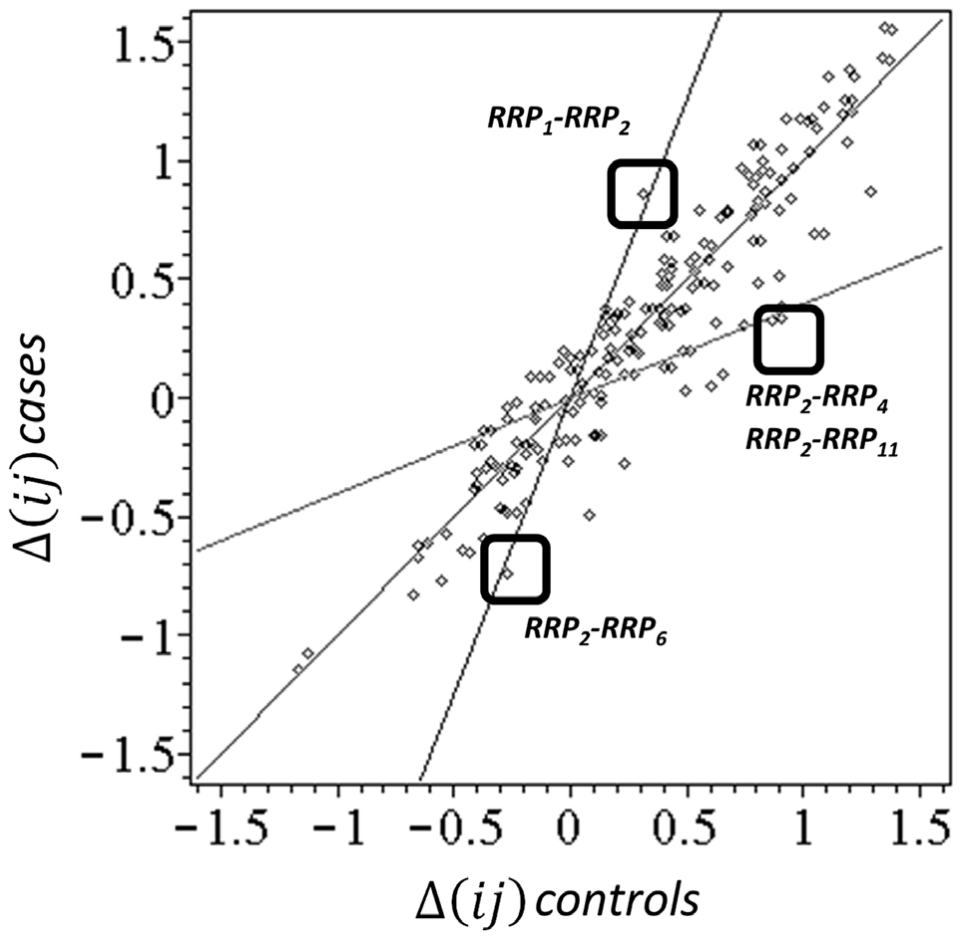
Selection of maximally case-control survival discriminating combination of distances from all *RRP*'s. Points are defined by the 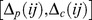 coordinates (see text) computed by averaging the distance differences over all patients separately in case and control sub-cohorts for all 190 possible ***RRP*** pairs. In the neighborhood of diagonal line 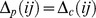 are non-discriminatory combinations. The two lines are used to identify the combinations, with maximal case – control and control-case bias in ***PRP***-***RRP*** distances. The optimal selection is shown by boxes.

An element of the 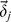 vector characterizes the distance of the personal CTLA-4 genotype pattern from reference, but does not include directionality and distances of the personal CTLA-4 genotype pattern from other reference patterns. To include that information into processed data, we therefore computed a complete set of 190 pairwise distance differences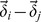, with ***i*** and ***j*** going through all 20 elements of the CTLA-4 differences from the four maximally case-control biased reference patterns ***RRP_i_–RRP_j_*** identified in **Fig.** **6**. These differences include directionality of the closeness of the personal genotype to one of the reference genotype patterns: 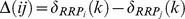 can be positive or negative. Assume that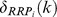  = −7,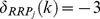.Then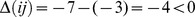. Thus, 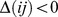 indicates that a personal CTLA-4 genotype profile is closer to ***RRj_i_*** while 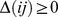 indicates that personal CTLA-4 genotype profile is closer to ***RRP_i_*** and 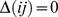 means that the personal CTLA-4 genotype profile has the same number of differences when compared either to reference profile ***RRP_i_*** or ***RRP_j_***. We computed the 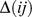 using distances from all 190 possible ***RRP***'s pairs, separately for cases and controls and averaged them for each sub-cohort, obtaining case mean 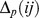 and control mean 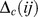 for each ***RRP***'s pair. Plotting these case and cohort averages against each other in the two-dimensional scheme allows direct identification of the reference CTLA-4 genotype pattern combinations that separate maximally the two sub-cohorts. For uniformly or randomly distributed CTLA-4 genotype pattern positions we obtain 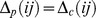 seen in the 2D plot as the diagonal ***y*** = ***x*** line. The combinations with maximal 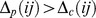 or 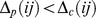, which are the desired clinically characteristic contexts will be in the 2D plot maximally distant from the diagonal. **Fig.** **6** shows the resulting 2D plot with the extreme combinations of the references indicated. The region of 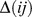 smaller than 0.5 is not considered, as there the subject's CTLA-4 genotype patterns are on average equally distant from both reference pairs.

**Figure 7:**
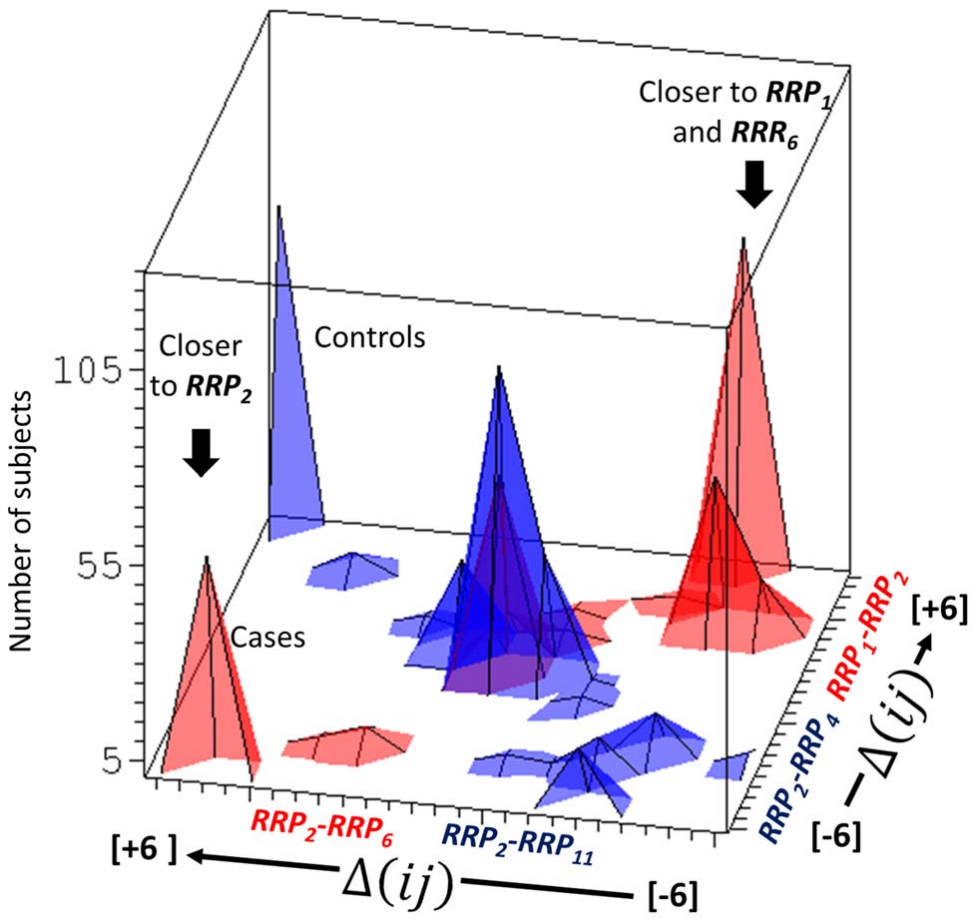
Histograms showing heterogeneity of distributions of individuals shown in the CTLA-4 genotype landscape, defined by the inter-personal differences in *prp*'s for the five most discriminating *RRP* combinations. Two selected combination of 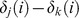 distance differences are plotted on ***x*** and ***y*** axes, on the ***z*** axis are numbers of subjects having a given combination of the distance differences. Blue-controls, red-cases.

**Fig. 7** shows histogram of patients with observed valued of 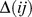. The patient or control distribution in the CTLA-4 genotype pattern space is not uniform or normal. We see clear heterogeneity: In both groups, there are three main patient subgroups. One, common for cases and controls has CTLA-4 genotypes equally different from all reference CTLA-4 allele relationships (central peak). Then there are two groups with their individual CTLA-4 genotype patterns significantly closer to one than to the other reference genotype relationship network.

**Figure 8:**
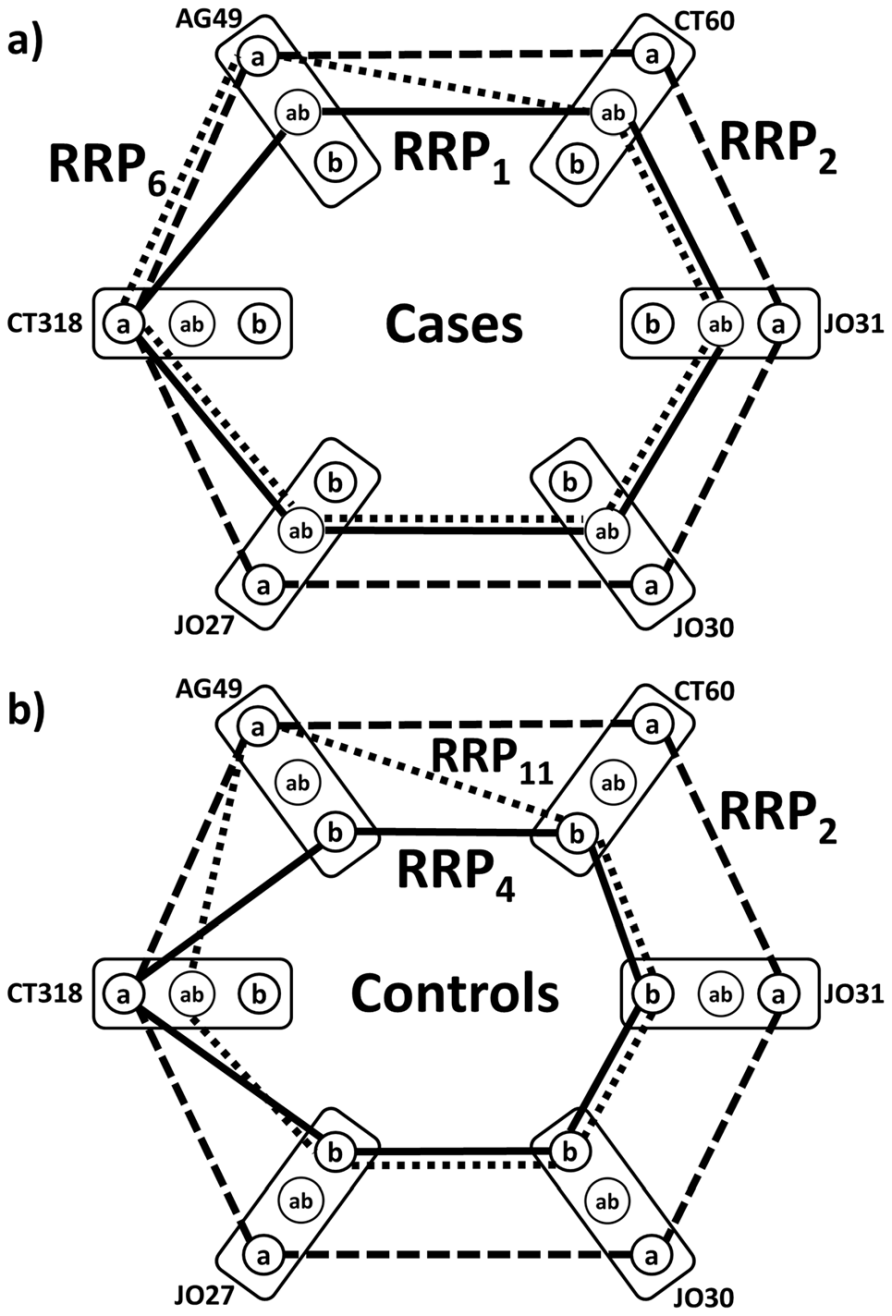
Comparison of CTLA-4 genotype relationship profiles of five most case-control discriminating *RRP*'s. ***RRP_2_*** (dashed edges) is shown in both panels for reference. Symbols as in Fig. 1.

**Fig. 8** shows the actual composition of these reference CTLA-4 genotype patterns for cases and controls. For controls, the dominant reference CTLA-4 genotype pattern is all major allele combination (***RRP_2_***) while for cases, ***RRP_1_*** dominates, where majority of studied CTLA-4 polymorphisms are in the heterozygous state. This heterogeneity might be utilized in focused prospective study of patients within the three subgroups identified: One being characterized by the minimally genetically affected CTLA-4, another having majority of CTLA-4 polymorphisms with heterozygous state and the third with mixed CTLA-4 genotype relationship patterns, equally different from the two extremes. It is clear that, contrary to melanoma patients, the healthy biosystem of controls can accommodate the CTLA-4 genetic variation where a majority of studied polymorphisms relate to the minor allele states that are identified as reference contexts for two groups with CTLA-4 genotype patterns different from “normal” ***RRP_2_***.

### Differentiation of the CTLA-4 genotype contexts between the long and short surviving sub cohorts of melanoma patients

Out of the 286 melanoma cases, we had 282 with survival data. Characterization of the possible differences between the long- and short-surviving patients now requires a different analysis strategy. First, we tested the choice of CTLA-4 genotype reference relationship patterns. After separate testing of results from NPS analysis of melanoma case CTLA-4 genotype relationship profiles, we found the simplest and statistically most significant results were obtained when the ***RRP_1_−RRP_20_*** resulting from the analysis of combined case/control cohort were used. That makes sense in light of previous standard statistical analysis indicating no significant differences in the actual CTLA-4 genotype patterns. A larger cohort combined from cases and controls provided better coverage of the possible reference CTLA-4 genotype relationship patterns. Moreover, the results were significant when the case sub-cohort was analyzed separately, and overlapped with the patterns identified using differences of distances from the combined analysis.

For the analysis of CTLA-4 genotype relationship pattern differences between the survival categories, we used a different strategy to make sure that what was found was indeed significant. We defined an overall survival threshold and separated the cohort into patients who lived longer or shorter than the selected threshold. We then ran the complete analysis described below and compared the statistical significance and performed logistic regression models to recognize the survival categories from the 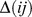. We systematically iterated through a threshold of 800 days to a threshold 1900 days, and found the optimal threshold at 1820 days (5 years). This threshold separated the cohort into balanced sub cohorts of 145 shorter and 137 longer surviving patients.

**Figure 9:**
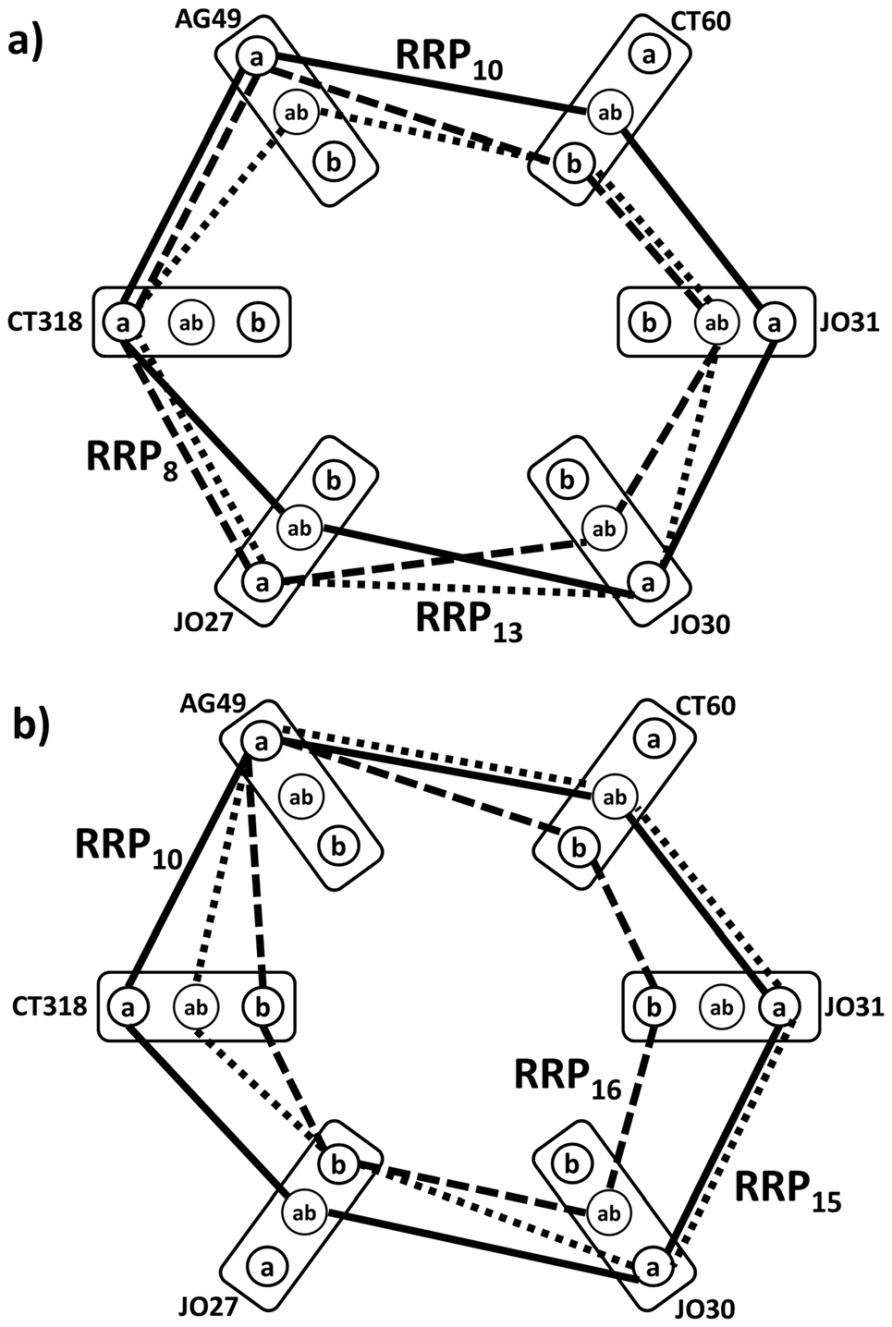
Comparison of *RRP*'s, distances from which are most significantly different in the two survival groups (overall survival longer or shorter than 5 years). ***RRP_10_*** is shown by solid edges in both panels (**a,b**) for reference.

We then computed the 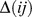 separately for both these survival-defined sub cohorts and tested the distributions of the results for all 190 CTLA-4 reference relationship pattern pairs. Out of the 190, only 4 combinations resulted in the statistically significantly different means of these distributions (see ***p***-value **Table** **2**). Here, ***RRP_10_*** reference pattern is the common context in all these CTLA-4 genotype relationship patterns, which are significantly biased between the longer and shorter surviving melanoma patients. Similar interpretation is now possible for the localization of the typical CTLA-4 genotype relationship patterns for these outcome different patients: For example, shorter surviving patients have typically positive 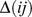 for ***RRP_8_***−***RRP_10_***, so they are closer to ***RRP_8_***, meaning that their CTLA-4 genotype tend to converge to 4 minor, one heterozygous and one major allele (see **Figure** **9**). Similar interpretation is possible for remaining significantly different genotype pattern pairs: ***RRP_10_***−***RRP_13_*** pairing have typically zero 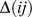 for shorter surviving patients, and positive for longer survivals, indicating that ***RRP_10_*** pattern with 4 major and 2 heterozygous alleles provides better functioning CTLA-4. Note that – contrary to genotype profiles with conserved allelic states of CTLA-4 polymorphisms – the CTLA-4 genomic profiles typical for cases-only cohort describe states of mixed allelic states of the six polymorphisms. For (CT318-AG49-CT60-JO30-JO27) profile, the ***RRP_8_*** has (**a-a-b-ab-ab-a**) allelic pattern, for ***RRP_10_*** it is (**a-a-ab-a-a-ab**) pattern.

**Table 2:** *p*-values for difference in mean difference distributions for distances of ***PRP***'s from ***RRP***'s pairs, differentiating two survival groups (longer and shorter than 5 years).

*RRP* combination	*p*-values
***RRP_8_***–***RRP_10_***	0.022
***RRP_10_***–***RRP_13_***	0.024
***RRP_10_***–***RRP_16_***	0.025
***RRP_10_***–***RRP_15_***	0.043

Similar interpretation is possible for remaining significantly different genotype pattern pairs: RRP10-RRP13 pairing have typically zero for shorter surviving patients, and positive for longer survivals, indicating that RRP10 pattern with 4 major and 2 heterozygous alleles provides better functioning CTLA-4. Note that – contrary to genotype profiles with conserved allelic states of CTLA-4 polymorphisms – the CTLA-4 genomic profiles typical for cases-only cohort describe states of mixed allelic states of the six polymorphisms. For (CT318-AG49-CT60-JO30-JO27) profile, the RRP8 has (a-a-b-ab-ab-a) allelic pattern, for RRP10 it is (a-a-ab-a-a-ab) pattern. These results allow characterization of the odds for overall survival shorter than 5 years for new patients with known status of six CTLA-4 SNP's. We implemented this computation into a Excel worksheet, which is available as Table S1, together with instructions for its use (Manual S1).

## Discussion

Using a novel approach to the analysis of SNP results for the CTLA4 gene, we have hypothesized that recognition of melanoma risk genotype profile requires an added dimension of analysis. This second step in the analysis progression moves from analyzing the means and variance of independent SNPs to analyzing the distributions of differences of individual CTLA-4 genotype profiles in the studied cohorts, in reference to normative reference profiles.

We argue that the observed haplotypes are the proper reference for this purpose, and that we need to use them to account for interpersonal variability in CTLA-4 genotype profiles. The approach generates 6-partite graphical depictions which are based upon algorithms that identified the same haplotypes and their frequencies in established statistical procedures. Importantly, this algorithm has shown that the haplotypes are not markers by themselves, but rather that their averaged constructs, identifying common co-occurrences of CTLA-4 SNPs in case and control cohorts are useful. Having both personal CTLA-4 genotype profiles and the normative reference co-occurring CTLA-4 SNP haplotype patterns represented by the ***K***-partite graphs has two main advantages:

### 

#### A

It determines from the data used to construct the g and through the decomposition algorithm we developed from the statistical conditions used in general characterization of haplotype the actual TOTAL number of haplotypes in the cohort (8 in both our cohorts). Considering that the theoretical number of haplotypes for ***g*** is 64, this is an important data reduction outcome of this approach. We know from other applications that in cases where deconstructed 6-partite graphs are close to random distributions of the conditional probabilities, the number of components needed to fully deconstruct the model increases significantly. Thus, small number of components in the ***g*** deconstruction implies the commonality/regularity in the CTLA-4 genotype profile composition and frequency in our study population. This is in agreement with the previous study results.

#### B

The component graphs ***rrp_i_*** are data-driven, information-rich references for exact quantitative computation of the 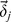 descriptors, which are tools enabling to change the focus of the analysis from means and averages to where we need it (i.e. towards differentiating features). Importantly, the ***rrp_i_***'s are **not** just mathematical constructs, but have well-defined genomic meaning, being haplotypes. This facilitates clinically relevant interpretation of the results in general and the individual (personalized) disease related markers in particular. Results validate the hypothesis.

Another important aspect of this work is its “translation” of the main molecular result of this paper to design of tools and algorithms that use the relationship-patterns between genotyped CTLA-4 variants to enable differential outcome analysis. Our approach allows to show, that in the relationship patterns picture of the individual CTLA-4 genotype, differential outcome can be caused by a “majority rule”, understood as a larger than critical deviation from an ideal, reference haplotype relationship pattern. Thus, same impact can be observed for different combinations of the personal CTLA-4 variants, which is clearly quantitatively captured in our NPS (relationship) based analysis, but causes problems in conventional approaches. This sharing of a certain level of differences from a reference normative pattern is very specific in relation to the kinds of patterns that share a particular property. This linkage of several heterogeneous patterns to one “functional” patient's individual difference is that other side of clinical data understanding, which can be brought to the plate using this approach.

Without the pattern-based approach, we would never recognize the relationship between those patterns and could not ask what is unique about them. More importantly, this common distance of personal CTLA-4 genotype profiles from reference genotype patterns may group patients that would conventionally not have been thought to be potentially grouped for interpretation. By definition, they have different patterns of CTLA-4 parameters, the conventional approach will tell you that these are different, so that you would never ask whether they have something in common.

Our approach – by contrast – has brought together patients with five different CTLA-4 genotypes so that we are forced to ask what these patterns have in common. We can now clearly identify that the **absence** of **one** common pattern from these five different, is what distinguishes cases and controls.

The combination of SNP's, shared by all individual patients' profiles that satisfy the condition of having the largest distance from one specific haplotype allows then discussing the mechanistic details in future studies (for example, why it is just this combination of major and minor allele in the 6 genotyped loci, which separates cases from healthy controls).

We also see how NPS helps in extracting collective properties of the CTLA-4 genotype through ***RRP***'***s*** characterizing different cohorts. In the processing of complete study data, i.e. from the subject set where about 50% are healthy controls, we observe clearly the dominance of “allelic uniformity” of the CTLA-4 landmarks (***RRP***'***s*** in **Fig.** **8**). On contrary, when only melanoma case sub-cohort is analyzed, the resulting characteristic CTLA-4 genotype ***RRP***'s patterns, that are separate the long and short survival categories (**Fig.** **9**) are indicating that for melanoma cases, the “allelic heterogeneity” dominates the functionally relevant CTLA-4 genotype status.

Key issue is that detailed characterization of the genotype by explicit consideration of the actual state of each SNP provides the significant clustering (for one survival group) or difference/distance (for the other survival group) of the ***prp***'s relatively to perhaps interesting and interpretable CTLA-4 genotype relationship patterns.

The limitations of this study are: (a) the size of the study cohort and (b) the number of the SNPs studied. Consequently, we did not fully exploit NPS to combine clinical and genomic information. However, this study was an effort at proof of principle for NPS and with this accomplished, these goals will now readily be undertaken. Specifically we will attempt to identify a priori, the compensatory and detrimental haplotypes through finding their function-related positive and negative descriptors.

**In summary:** Pattern based polymorphism relationship analysis revealed that in healthy controls, the context in which the CTLA-4 and its genetic variants operates is compatible with the genotype with relationship pattern with “consensus” alleles in all six sites. While we see some relationship pattern differences between long and short overall survival groups, these are not independently recognized, we need to know who is long and who is short surviving. To obtain really independent, statistically significant, prediction of the long or short survival we thus need to go one additional step: consider that there is coherence pattern between assayed regions of CTLA-4 gene and that this coherence pattern is affected by the polymorphisms in the personal genotype in exactly computable way. This is provided by the categorization of disease outcome via analysis of thermodynamic changes in the in CTLA-4 SNPs discovered by entromics [19], and quantified by the differences in matrices that quantify the energy weights associated with the various genotype profiles in individual patient entromic coherence networks.

## Supporting Information

Figure S1 **Iterative algorithm steps involved in decomposition of study graph** ***g*** **into** ***rrp*** **'s.** Shown are residual graphs after greedy removal of respective reference relationship patterns in the order of their decreasing multiplicity.
(TIF)

File S1 **Algorithm for the identification of haplotypes.**
(DOC)

Manual S1 **Instructions for using the Excel worksheet implementation of CTLA-4 allelic patter based survival category prognosis model for melanoma patients.**
(DOC)

Table S1 **Excel worksheet implementation of the categorization of melanoma patients into shorter/longer survival subgroups using distances between personal and reference allelic patterns of six CTLA-4 polymorphisms studied in this paper.**
(XLSX)
