# Supplementary figures and images for: A New Mathematical Model for the Interpretation of Translational Research Evaluating Six CTLA-4 Polymorphisms in High-Risk Melanoma Patients Receiving Adjuvant Interferon
Source: PLoS One. 2014 Jan 27;9(1):e86375. doi: 10.1371/journal.pone.0086375 (PMC3903519; doi:10.1371/journal.pone.0086375)

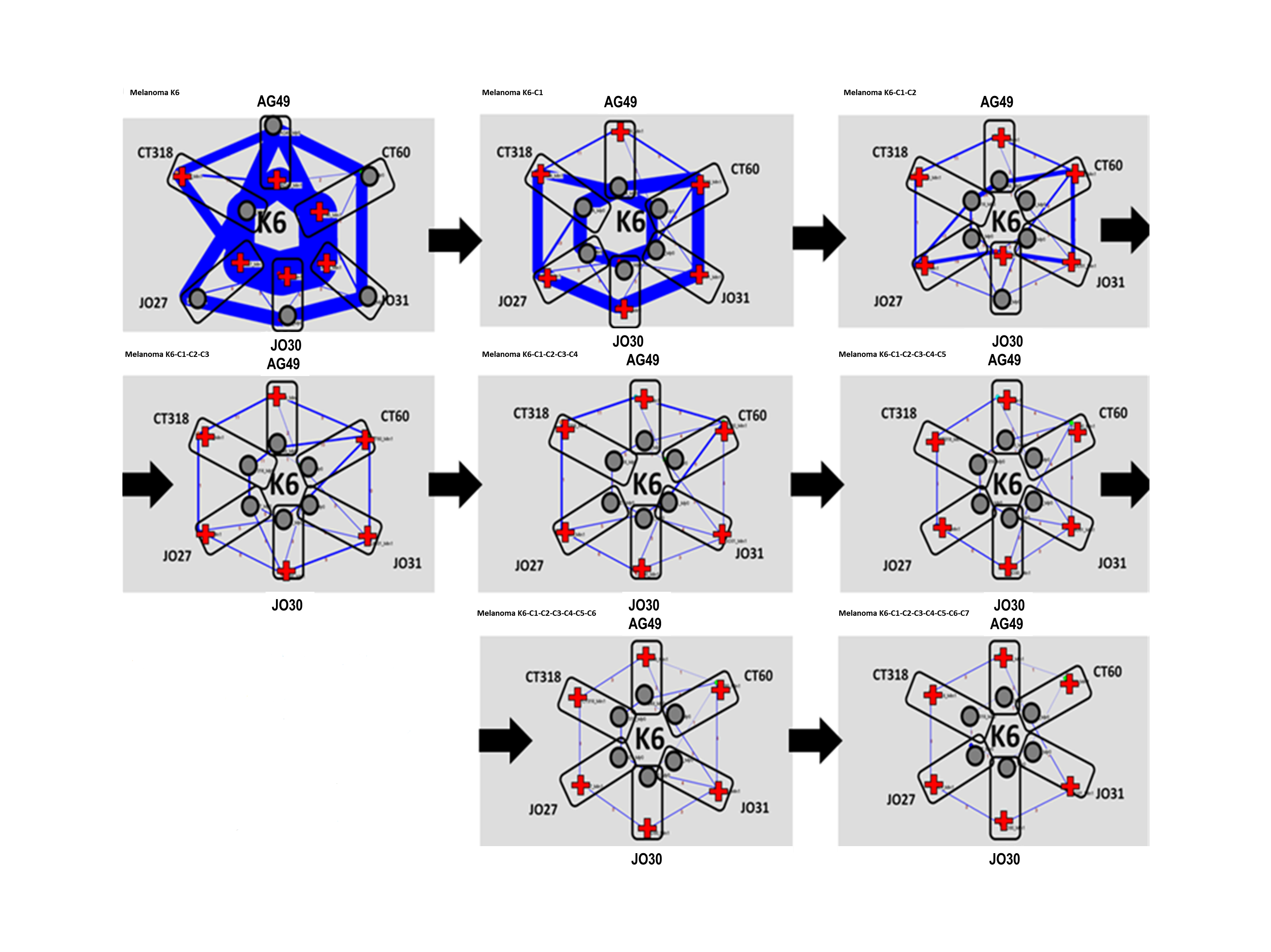

Supplement: Figure S1 — Iterative algorithm steps involved in decomposition of study graph g into rrp 's. Shown are residual graphs after greedy removal of respective reference relationship patterns in the order of their decreasing multiplicity. (TIF) [file pone.0086375.s001.tif]
