# Supplementary material for: A New Mathematical Model for the Interpretation of Translational Research Evaluating Six CTLA-4 Polymorphisms in High-Risk Melanoma Patients Receiving Adjuvant Interferon
Source: PLoS One. 2014 Jan 27;9(1):e86375. doi: 10.1371/journal.pone.0086375 (PMC3903519; doi:10.1371/journal.pone.0086375)
Supplement: File S1 — Algorithm for the identification of haplotypes. (DOC) [file pone.0086375.s002.doc]

**File S1:**

The algorithm for identifying the presence and relative frequency of all haplotypes in a genotyped cohort (see **Fig. S1**):

1. In the complete ***g***, identify the 6 SNP alleles represented by the cycle ***C*_max_** with the highest sum of all 6 edge weights.
2. In ***C*_max_**, find the edge with the minimal weight ***W*_min_** out of all 6.
3. From ***g***, remove the cycle ***rrp_i_***, which has all edges with weight ***W*_min_**. Vertices, connected by edge in ***rrp_i_*** define the molecular composition of this haplotype, ***W*_min_** is the frequency of this haplotype.
4. The remaining subgraph $g^{i}$ is again subjected to steps 1-3 above, until all edges from ***g*** are removed. This generates the series of all haplotypes ***rrp_i_*** (***i***=1..***k***) in the cohort, together with their frequencies (***W*_min_(i)**, ***i***=1..***k***).
