# Supplementary material for: A New Mathematical Model for the Interpretation of Translational Research Evaluating Six CTLA-4 Polymorphisms in High-Risk Melanoma Patients Receiving Adjuvant Interferon
Source: PLoS One. 2014 Jan 27;9(1):e86375. doi: 10.1371/journal.pone.0086375 (PMC3903519; doi:10.1371/journal.pone.0086375)
Supplement: Manual S1 — Instructions for using the Excel worksheet implementation of CTLA-4 allelic patter based survival category prognosis model for melanoma patients. (DOC) [file pone.0086375.s003.doc]

MANUAL S1

The instructions for the future user is:

1. Enter the patient's personal genotype by indicating the correct alleles by "1" and replacing all other "1"s by "0" in the input line 3 as indicated.

2. The distances of personal relationship profile (describing the actual CTLA-4 haplotype) from the RRP's 8,19,13,15 and 16  are automatically shown on line 6, columns A-E (results should be negative integers. If decimal numbers appear, there is an error in the entered genotype data). The distance differences, relevant for survival categorization (see Table 2 and text of the paper),  are shown on line 6, columns G-J.

3. The classification of a patient to the survival group (longer or shorter than 5 years) is identified in the distance map by X (if the CTLA-4 haplotype is unique for the survival group, yellow or red cells respectively) or by the numerical value of hazard ratio of being in the short surviving group (in blue cells, see legend). The hazard ratio shown is computed from the data of the study used in this paper.
